# First recorded birth of a giant armadillo (*Priodontes maximus*) under human care and notes on fetal characteristics and neonatal care

**DOI:** 10.29374/2527-2179.bjvm000325

**Published:** 2025-09-23

**Authors:** Carlos Andrés Aya-Cuero, Yully Alejandra Pardo-Moreno, Laura Katerine León-Aguilar, Pablo Felipe Cruz-Ochoa, Ana Raquel Gomes Faria, Maria do Livramento Barros de Oliveira, Clarissa Machado de Carvalho, Arnaud Leonard Jean Desbiez

**Affiliations:** 1 Fundación Kurupira, Bogotá, Colombia.; 2 Instituto de Turismo del Meta - Bioparque Los Ocarros, Villavicencio, Colombia; 3 Fundação Jardim Zoológico de Brasília, Candangolândia, Brasília, DF, Brazil.; 4 Hospital Veterinário, Setor de Animais Silvestres, Universidade de Brasília, Brasília, DF, Brazil.; 5 Instituto de Conservação de Animais Silvestres (ICAS), Campo Grande, MS, Brazil.

**Keywords:** Chlamyphoridae, reproduction, neonate size, Orinoco region, Xenarthra, Chlamyphoridae, reprodução, tamanho neonatal, região Orinoquia, Xenarthra

## Abstract

Information on breeding under human care is essential for conservation efforts, particularly for endangered species such as the giant armadillo (*Priodontes maximus*). Key aspects of its reproductive biology, such as neonate size, weight, and early development, remain largely undocumented. This study presents two case reports of *P. maximus* neonates and one of an unborn individual. The first documented birth of a giant armadillo under human care was documented in Colombia, where a pregnant female was rescued from the wild. We describe details of this neonate, along with observations of an unborn fetus found in the eastern Colombian plains and the artificial hand-rearing of a neonate rescued in Tocantins, Brazil. The information presented here offers valuable insight for future efforts to breed and maintain this elusive species under human care, an increasingly important strategy for conservation and for understanding aspects of its natural history.

## Resumo

Informações sobre a reprodução em cativeiro podem ser fundamentais para os esforços de conservação, especialmente no caso de espécies ameaçadas de extinção, como o tatu canastra (*Priodontes maximus*). Este estudo descreve dois relatos de casos de tatus canastra recém-nascidos e outro sobre um indivíduo ainda não nascido desta espécie esquiva de tatu. O primeiro nascimento documentado de um tatu canastra sob cuidados humanos foi documentado na Colômbia, esta fêmea foi resgatada grávida da natureza. Descrevemos detalhes desse neonato, juntamente com notas sobre um caso isolado de um feto encontrado morto nas planícies do leste colombiano e os esforços de criação artificial à mão de um tatu-canastra juvenil resgatado ainda filhote no Tocantins, Brasil. As informações aqui documentadas são cruciais para futuras tentativas de criação e manutenção de tatus carreta indivíduos sob cuidados humanos, uma ferramenta cada vez mais importante para os esforços de conservação de espécies e até mesmo para o conhecimento dos padrões da vida selvagem.

## Introduction

The giant armadillo *Priodontes maximus* (Kerr, 1792) is the largest living armadillo, reaching up to 150 cm in length (including the tail) and weighing 28-50 kg. It is a specialized insectivore with solitary, nocturnal, and fossorial behavior (Carter et al., 2016; Desbiez et al., 2019, 2021). It requires large home ranges (up to 25.18 km^2^) and has low population density and reproductive rates (Carter et al., 2016; Desbiez et al., 2020c). The species is endemic to South America (Carter et al., 2016). It is classified as vulnerable in Colombia, Brazil, and globally (Chiarello et al., 2015; Colombia, 2024; Superina et al., 2025) and is included in Appendix I of the Convention on International Trade in Endangered Species of Wild Fauna and Flora (2024). Despite facing multiple anthropogenic threats, basic reproductive information remains scarce, including its mating season and offspring care in natural conditions or under human care (Aya-Cuero et al., 2015). Known reproductive traits include a single pup per birth, a gestation period of approximately five months, and lactation lasting 6-8 months (Desbiez et al., 2020a). The offspring stays near the mother for at least 11-12 months and continues using her burrow until about 18 months (Desbiez et al., 2020a). Even after weaning, the pup may remain in the maternal territory for several years (Aya-Cuero, 2023; Desbiez et al., 2020a). Sexual maturity is estimated to occur between 6.5 and 9 years of age (Luba et al., 2020).

Information on breeding under human care is critical for conservation, especially for endangered species (Wakchaure & Ganguly, 2016). Current knowledge on giant armadillo offspring in the wild is limited to two studies using camera traps placed at burrow entrances, capturing pups only after they were strong enough to emerge, around 25 days old (Desbiez et al., 2020a). In the Colombian Orinoquia, three females with a single pup each were recorded between 2013 and 2014 (Aya-Cuero et al., 2015), whereas seven reproductive events were monitored through radiotelemetry and camera traps in the Pantanal of Brazil between 2010 and 2017 (Desbiez et al., 2020b). Under human care, the only documented case involves a female neonate rescued at approximately seven days old and taken to the Brasília Zoo, where she became the first known individual of the species successfully raised in such conditions (Ana Raquel, Pers. Comm). Some aspects of her management and breeding were described by Kluyber & Desbiez (2023), and additional details are provided here. The individual died at five years of age. To date, the size and weight of *P. maximus* at birth remain undocumented in both wild and human care settings.

## Case descriptions

### Case 1: Birth in Colombia

On September 7, 2023, the environmental authority Cormacarena, via its wildlife ambulance, rescued an adult female *P. maximus* weighing 41 kg, with a head-body length of 94 cm, tail length of 56 cm, abdominal circumference of 101 cm, and thoracic circumference of 87 cm. She was found in Puerto Rico, Meta, dehydrated, weak, and prostrate, with a wound suggestive of a snake bite. On admission, clinical examination revealed epistaxis, pale vulvar mucosa, and dyspnea; lung auscultation detected stridor.

As part of the diagnostic workup, a thoracic ultrasound was performed using Mindray^®^ DP-50 equipment to assess internal organs and detect free fluid. No signs of pregnancy were observed, and the female was not considered pregnant. Radiological studies conducted on September 8, 2023, at the veterinary clinical center of Universidad de Los Llanos focused on the skull and thorax to rule out bone lesions; the abdomen was not examined. A follow-up ultrasound using Chison^®^ Ebit 30 equipment also showed no evidence of pregnancy. Unfortunately, images from both the ultrasound and radiographs were not saved. After 28 days, the individual regained physical condition and, as of April 17, 2025, resides in good health at Bioparque Los Ocarros.

The pregnant female was housed with another adult female in a 12 m^2^ exhibition area that included two 4 m^2^ management rooms. These rooms had cement floors covered with dry straw for resting. Another section featured a sand substrate, two shallow artificial caves, and a water pool. The entire area was permanently covered with synthetic material to prevent direct sunlight exposure. The female was fed 1000 mL of a semiliquid mixture twice daily at 9:00 and 16:00, containing water, chicken eggs, Pet Milk^®^, Ensure^®^, apple, Nestum Cereal^®^, serum protein, alfalfa pellets, and peat. Additional supplements included 100 g of boiled eggs with a Canapet scoop, and environmental enrichment consisted of flavored yogurt with small pieces of banana, peach, and watermelon.

**Figure 1:**
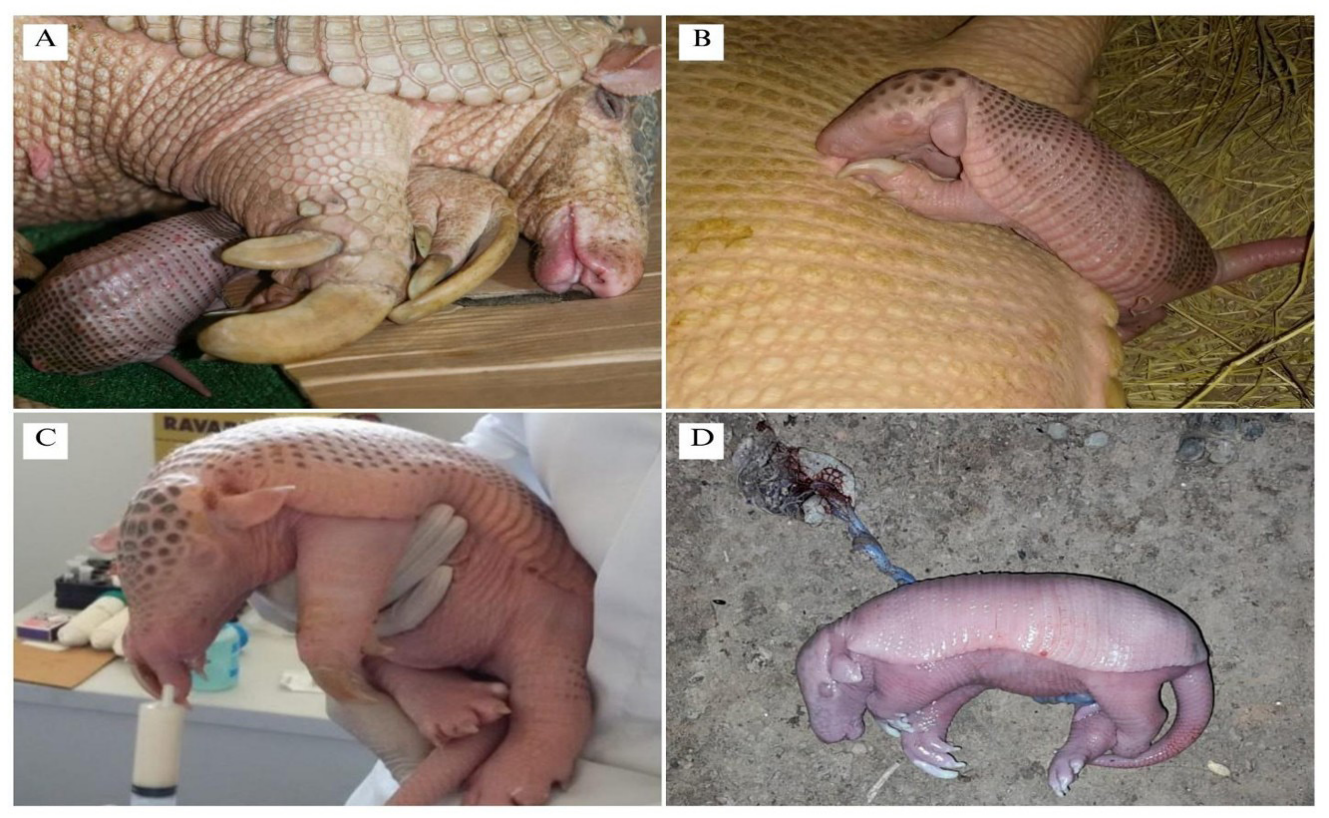
Photographs of *Priodontes maximus* neonates and an unborn individual. (A) and (B) Female neonate suckling at Bioparque Los Ocarros; (C) *Priodontes maximus* pup on the day of its arrival at Brasília Zoo, Brazil, showing closed eyes, drooping ears, and soft, lightly pigmented carapace plates; (D) Unborn individual extracted from the mother following hunting for subsistence in Vichada, Colombia. Photograph by Henry Gutiérrez and Instituto de Turismo del Meta Communications Team.

Forty-eight days after her arrival at Bioparque Los Ocarros, the adult female exhibited vulvar mucus and bleeding. Ten days later, on November 13, 2023, at 17:28, she gave birth to a female neonate weighing 542 g and measuring 49.4 cm in total length. The birth occurred in a cave-type shelter within the giant armadillo exhibit area. While the mother slept elsewhere, the neonate was weighed and measured. Found approximately two meters from the mother, the neonate showed altricial morphological (closed eyes, no hair) and behavioral traits (limited mobility, absent sucking reflex; Figure 1A; Table 1). Its carapace and cephalic shield displayed brown pigmentation in well-defined circular patterns. The umbilical cord detached on the second day after birth. No nesting behavior was observed before or after parturition.

**Table 1:** Characteristics and body measurements of unborn, neonate, and juvenile *Priodontes maximus* individuals recorded under human care or observed in the wild in Colombia and Brazil.

**Body measurements**	**Unborn 1 “Vichada”**	**Neonate 1 “Bioparque”**	**Pup 2 “Mabu”**
Morphometric measurements at birth or arrival			
Body mass	-	0.542 kg	2.8 kg
Head-tail length	-	49.4 cm	61.63 cm
Tail length	-	14 cm	23.43 cm
Arm length	-	6 cm	-
Hand length	-	3 cm	9.53 cm
Leg length	-	1 cm	-
Foot length	-	5 cm	11.33 cm
Ear length	-	1.9 cm	3.13 cm (right ear) 3.25 cm (left ear)
Head length	-	4.5 cm	11.33 cm
Head width	-	8.2 cm	-
Head circumference	-	-	19 cm
Characteristics			
Estimated life	3-4 months into gestation	4-5 months into gestation	5 months gestation and 7 days old
Sex	Indeterminate	♀︎	♀︎
Pigmentation of the carapace	Absent	Present	Present
Umbilical cord	Present	Detached after two days	Absent
Eyes	Closed	Closed	Closed at arrival, open after 40 days
Rectal temperature	-	35.2-36.3 °C	35 °C
Appearance of carapace osteoderms	Any kind of pigmentation, all pink and flexible	Flexible, mostly pink	Flexible, mostly pink, with dark pigmentation at the center
Drooping ears	Present	Present	Drooping at arrival, erect after 40 days

The adult female showed no nursing, protective, or defensive behavior toward her offspring (Figure 1B). To encourage the neonate to locate the nipple, the mammary glands were stimulated by milking, and the extracted milk was smeared on the nipple to activate foraging behavior via scent. This approach aimed to optimize initial colostrum intake and support offspring viability. During the first two days, the nipple was stimulated every three hours, and the pup was placed nearby to feed, resulting in a weight gain of 110 g within the first 48 hours. A video of this behavior is available at YouTube (2025).

After the first three days, the newborn began to lose weight, and suckling behavior declined. Assisted rearing and medical evaluation were initiated. However, after 24 hours without suckling, its health worsened. The newborn died on November 17, 2023, at 9:25, four days after birth. Given the poor physical condition and absence of head movement, starvation was initially suspected as the cause of death. Autopsy revealed hemorrhagic necrotic hepatitis associated with severe omphalophlebitis, along with hemorrhagic adrenalitis and severe gastroenteritis, primarily affecting the duodenum and jejunum. These findings indicate septicemia, likely resulting from bacterial entry via the umbilicus or oral route, leading to the death of the newborn.

### Case 2: Neonatal rescue and rearing in Brazil

On October 3, 2014, a wild *P. maximus* pup was admitted to the Wild Animal Sector of the Small Animal Veterinary Hospital at the University of Brasília, Federal District, Brazil, via referral from the Wild Animal Screening Center (CETAS-DF). According to the CETAS-DF report, a member of a land excavation team on a farm in Tocantins, Brazil voluntarily brought in the pup. The excavation had exposed a burrow containing an adult and a pup; the adult fled and did not return. The pup remained motionless in the damaged burrow for several hours. When the work ended and the adult had still not returned, the team concluded that the pup was still dependent and decided to remove it. The individual who delivered the pup lived near CETAS-DF, facilitating its transfer.

A clinical assessment confirmed that the pup was a female, with closed eyes and a slightly raised belly button, suggesting recent loss of the umbilical cord. She had an excellent body score (3/5 on a five-point scale where 1 = thin and 5 = obese), weighed 2.8 kg, was well-hydrated, had a body temperature below 34 °C, and was highly reactive to manual restraint. She appeared hypothermic, showing torpor, limited movement, weak responsiveness to stimuli, and subnormal temperature. According to MacNab (1985), adult armadillos have a body temperature approximately 2 °C lower than other mammals and limited thermoregulatory capacity. Moreover, newborn and infant mammals cannot thermoregulate effectively and require parenteral care to stabilize temperature. Once warmed and reaching 35.4 °C, the pup responded better to stimuli and fed more efficiently with milk replacer. Her carapace remained soft on palpation, with poorly pigmented horny plates and thin, rosy skin. Based on her immature features, including an open ear canal, drooping ears, and closed eyes (Figure 1C), birth was estimated around September 28, 2014.

The following day, basic physiological parameters were recorded: rectal temperature (35.4 °C), heart rate (144 beats per minute), and respiratory rate (100 breaths per minute). While she was sleeping, a general examination was performed, during which a tick (*Amblyomma parvum*) and several nymphs were removed from her abdomen. Petechiae were observed on the body, likely caused by ectoparasites. Fipronil (Frontline^®^ Topspot; Merial, Lyon, France) was applied to the back based on body weight. The first urination occurred the morning after arrival, with a strong odor and translucent color. Defecation occurred in small quantities two days after arrival, and the feces were dry.

The pup was housed in a warm environment inside a plastic box lined with cloths to simulate a comfortable space. The box was selected for ease of cleaning, portability, and its ability to restrict movement and provide environmental control. To keep body temperature within the ideal range (26-27 °C, with humidity above 60%), a space heater was used, alone or in combination with a thermal bag containing hot water placed under the cloths, particularly on colder days. During the first 60 days, ambient temperatures averaged 20-25 °C, requiring continuous heating. After this period, temperatures rose, and the pup began maintaining body temperature independently, reducing the need for external heat sources.

The pup was on a once-daily liquid diet of Pet Milk^®^ throughout this period, using a specific feeding method for hand-rearing. The traditional vertical bottle-feeding position used for other mammals can cause aspiration in giant armadillos. Instead, an adapted bottle with a thin elastic tube was used in a ventral decubitus position, which proved successful and is recommended. During nursing, the pup experienced varying degrees of diarrhea with significant gas, followed by episodes of constipation. Weaning began at 22 weeks of age, prompted by chronic diarrhea, which made a dietary change necessary. The new diet included 20% Royal Canin Gastrointestinal Loaf Canned Dog Food, 30% cooked ground beef, 20% fruits (banana, apple, papaya, guava, mango), 25% cooked vegetables (sweet potato, pumpkin, carrot), 1% sugarcane fiber, and 3% calcium carbonate. It was offered once daily at night.

### Case 3: Unborn individual found in Vichada, Colombia

At 21:00 on a night in February 2021, during the dry season in the Llanos region, local inhabitants in the rural area of La Primavera, Vichada, hunted an adult female giant armadillo in a riparian forest for subsistence. An unborn fetus was later found inside the mother (Figure 1D). The case was documented by Henry Gutiérrez on the iNaturalist platform with support from Fundación Omacha, a local non-governmental organization, through community monitoring under the Llanos Armadillo Conservation Program (iNaturalist, 2025; Superina et al., 2023). Unfortunately, limited information was available, as only a photograph and basic details from local residents were accessible.

## Discussion

All reported cases occurred incidentally and were not part of a study targeting offspring or pregnant females. The cases align with existing literature indicating a litter size of one. Although the possibility of two offspring has been suggested, this was based on an old photograph showing two individuals of different species (Desbiez et al., 2020a).

In terms of size, neonate 1 measured 1.4% of average adult body mass, assuming a typical adult weight of 35 kg for this species, lower than previously reported. Superina and Loughry (2012) noted that most armadillos are born at 6-7% of adult mass, and based on this, Aya-Cuero et al. (2015) estimated a birth weight of 1.9-3.5 kg. This discrepancy supports the possibility of abortion or premature birth, which may explain rejection by the female and early death of the neonate. Neonates in the first and second cases exhibited features described by Desbiez et al. (2020a), including light coloration, closed eyes, and a soft carapace. This soft carapace, also noted in armadillo fetuses by Vickaryous and Hall (2006) and Superina and Loughry (2012), is more flexible because the osteoderms are small and separated by loose connective tissue. Given the limited reproductive data, the pup born at Bioparque appeared slightly larger than the unborn individual in the third case. In that case, the carapace and cephalic shield lacked pigmentation, whereas these characteristics were already developed in the first neonate.

The reported cases confirm that births occur during the dry season. In Meta and Vichada departments of Colombia, the dry season spans November to March. In Tocantins State, Brazil, it runs from May to October. The record in the first case supports this pattern, as November marks the start of the local dry season, and the Bioparque pup was born in that month. Even if the birth was premature, a full-term gestation would still fall within the same season. In relation to the third case, the hunting and consumption of *P. maximus* in the Llanos region was documented by Aya-Cuero et al. (2021). However, the impact of this practice on reproductive output remains unclear, particularly in a threatened species with a low reproductive rate. Given the timing during the reproductive season and the extremely slow population growth of the species, hunting likely has a substantial negative effect on population viability.

In the first case, we hypothesize that the pregnant female arrived at Bioparque Los Ocarros two to three months into gestation, based on the neonate being born approximately two months later. However, a premature birth cannot be ruled out given the small size of the newborn. In addition, whether *P. maximus* exhibits embryonic diapause, like other armadillo species such as *Dasypus novemcinctus* and *D. hybridus* (Fenelon & Renfree, 2018), remains unconfirmed, and the gestation period may vary depending on conditions. To date, no evidence of this phenomenon has been recorded in species within the Chlamyphoridae family.

The signs of pregnancy in giant armadillos were not obvious. False negatives on ultrasound likely resulted from the focus on vital organs, such as the kidneys, bladder, liver, and spleen, during examination, rather than on gestational assessment. Moreover, the gestational development of the species remains undocumented. Although limited in this case, ultrasonography remains a widely recommended diagnostic tool (Han et al., 2024) and is considered safe and non-invasive for pregnancy detection in both domestic and wild animals, as it avoids ionizing radiation associated with X-rays, which may negatively affect fetal development (Mohsin et al., 2023). According to Celik et al. (2022), infections are well-documented causes of early neonatal death in wildlife. Sepsis, in particular, is a leading cause of neonatal mortality in veterinary medicine (Celik et al., 2022) and highlights the need for careful management of births under human care. The naturally low metabolic rate of armadillos and immunodeficiency in newborns may increase susceptibility to vertical and horizontal infection. In both wild animals and humans, neonatal sepsis presents with nonspecific signs, requiring confirmatory testing for diagnosis (Celik et al., 2022). Additional diagnostic exams are therefore necessary.

Notably, the newborn showed depigmentation on the cephalic shield and carapace, along with an abscess emerging from a small area of the carapace, possibly linked to demineralization or structural weakening. Superina and Loughry (2012) discussed the role of the carapace in reproduction, including mineral mobilization to meet the demands of fetal development and lactation. In studies of other Chlamyphoridae species, such as *Zaedyus pichiy*, no evidence of demineralization was found across reproductive stages, including pregnancy. However, high bone mineral density may be critical for supporting reproduction in this species (Actis et al., 2017). In insectivorous species, substantial maternal calcium investment may be essential for reproduction, as insects are a poor calcium source (Superina & Loughry, 2012). Although pregnancy-related depigmentation cannot be ruled out, further studies are needed to clarify this association.

Reproduction in giant armadillos remains poorly understood, with many knowledge gaps resulting from the difficulty of locating the species in natural habitats and the limited number of researchers working on this taxon. Animals under human care offer valuable opportunities to gather reproductive data. Comizzoli et al. (2000) noted that for endangered species that have never reproduced under human care, such as the giant armadillo, reproductive biotechnologies including insemination and cloning, may provide useful tools. In this context and in future incidental encounters, the present report offers initial recommendations for caring for neonates. Future research should focus on reproductive aspects.

## Conclusion

The neonates of *P. maximus* are extremely delicate and require specialized care distinct from protocols used for other mammals. Understanding basic information on morphology, breeding, and neonatal care in endangered species is essential to identify their needs and the factors that influence development into adulthood. The information presented here is vital for future efforts to maintain individuals under human care, an increasingly important conservation tool, and for improving knowledge of wildlife behavior and reproductive and ecological patterns. Documenting births under human care also provides rare insights into fetal development and neonatal care, which are nearly impossible to observe in the wild, particularly in cryptic species such as giant armadillos.
